# What did inspire me to write the abstract for the EPMA Congress?

**DOI:** 10.1186/1878-5085-5-S1-A70

**Published:** 2014-02-11

**Authors:** Tatjana Josifova

**Affiliations:** 1 Ophthalmological Clinic, University of Basel, Switzerland

**Keywords:** diabetic retinopathy, vision loss, treatment strategies, stem cells, diabetic complications, blindness, eye screening, personalized treatment

## Case Report

Type 1 Diabetes Mellitus (DM)

**Figure 1:**
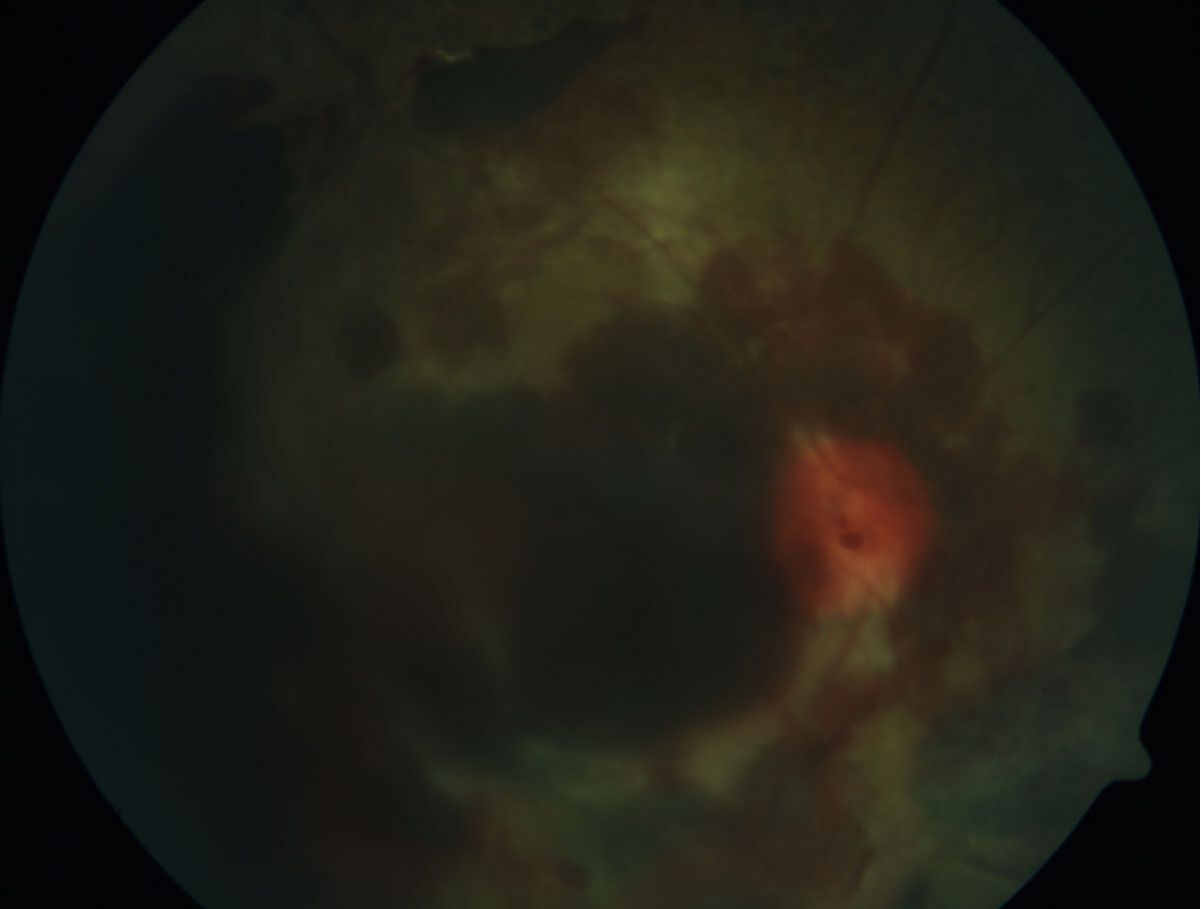
Fundus photography of huge retinal bleeding and retinal detachment in PDR

Male 22 years of age (Figure 1)

Insulin dependent since 2003

Visual acuity:

OD: 0,10

OS: Light Perception

Preparing for surgical treatment

Operation rejected because of:

Blood hypertension (230/110 mmHg)

HBA1C: 10.7 %

Anemia

Renal Insufficiency (not diagnosed)

Hart ischemic changes, left ventricular hypertrophy (not diagnosed)

DM is a major cause of avoidable blindness all over the world. Patients with diabetic retinopathy (DR) are 25 times more likely to become blind than non-diabetics. Prevalence of type 1 diabetes is 10-15% of the diabetic population. Prevalence of DR in Wisconsin Epidemiological Study of Diabetic Retinopathy was 50.1 and 54.2% in the diabetes control and complications trial in type 1 diabetes [1] and 35-39% in United Kingdom Prospective Diabetes Study in type 2 diabetes [2]. In two studies from India, the prevalence of DR in type 2 diabetes patients were 34.1% and 37% [3].WHO estimates - India has 31.7 million diabetic subjects at present. In USA, DR is the leading cause of new blindness in people 25-74 years of age. Approximately 700,000 persons have proliferative DR (PDR). Each year another 65,000 are diagnosed with the condition. A recent study has estimated that among people with diabetes aged 40 or older, 28.5% will develop DR. Worldwide 20 million people have PDR (very severe eye complication), with this number projected to increase to over 30 million by 2030. After 10 years of onset of DM, blindness was 1.8, 4.0 and 4.8% in type 1, insulin-treated type 2 and non-insulin-treated type 2 patients, respectively. In these three groups of patients, the 10-year incidence visual impairment (loss of 15 letters on a scale of 0-70 letters) was 9.4, 37.2 and 23.9%, respectively [4]. Good glycemic control arrests the development and progression of DR, but still there are a lot of factors that can influence the DR visual loss. Technological advances have improved the diagnostic accuracy of screening methods and given an access to the diabetic patients to the specialist care. In the last decades, the treatment strategies have been revised and besides laser photocoagulation, include surgical interventions and pharmacotherapies. DR will often go unnoticed when the condition is in its early stages but advanced symptoms can include blurred or sudden vision loss. Diabetics are advised to take advantage of the eye screenings which are offered free of charge in the UK, to detect any signs of the disease. Stem cell research, especially the embryonic one, holds great promise in the search for a cure for type 1 diabetes and provide a powerful tool for controlling type 2 diabetes [5]. The first results of the stem-cells DR- treatment were presented at the ARVO in 2011. Patients experienced improvements in the function of the optic nerve, blood flow to the eye and reduction of inflammation in the six-month period following injection and became stable for the entire follow up period. To achieve our goals of personalized treatment, we need EU support in different projects that will connect the physicians with basic science investigations and biotechnology. In the overall economic downturn tremendous budget cuts will only lead to huge burden of diabetic complications treatment.
